# ESR EuroSafe Imaging and its role in promoting radiation protection – 6 years of success

**DOI:** 10.1186/s13244-020-00949-5

**Published:** 2021-01-07

**Authors:** Guy Frija, Christoph Hoeschen, Claudio Granata, Eliseo Vano, Graciano Paulo, John Damilakis, Lluis Donoso, Lorenzo Bonomo, Reinhard Loose, Steve Ebdon-Jackson, the European Society of Radiology

**Affiliations:** 1 Paris Descartes University, Paris, France; 2 Institut Für Medizintechnik, Otto-Von-Guericke Universität, Magdeburg, Germany; 3 Department of Paediatric Radiology, Institute for Maternal and Child Health - IRCCS “Burlo Garofolo”, Trieste, Italy; 4 Radiology Department (Medical Physics), Complutense University, Madrid, Spain; 5 ESTESC - Coimbra Health School, Medical Imaging and Radiotherapy Department, Instituto Politécnico de Coimbra, Rua 5 de Outubro, S. Martinho Do Bispo, Coimbra, Portugal; 6 School of Medicine, University of Crete, Iraklion, Crete, Greece; 7 Department of Medical Imaging, Hospital Clínic of Barcelona, University of Barcelona, Barcelona, Spain; 8 Catholic University of Rome, Rome, Italy; 9 Institute of Medical Physics, Nürnberg, Germany; 10 Medical Exposure Regulatory Infrastructure Team, CRCE, Public Health England, Chilton, Didcot, UK; 11 Am Gestade 1, Vienna, Austria

**Keywords:** Radiation protection, Dose management, Safety standards

## Abstract

This article introduces the European Society of Radiology’s EuroSafe Imaging initiative in the year of its 6th anniversary. The European and global radiation protection frameworks are outlined and the role of the EuroSafe Imaging initiative’s Call for Action in successfully achieving international radiation protection goals as set out by those frameworks is detailed.

## Patient summary

Six years ago, the European Society of Radiology created its EuroSafe Imaging Initiative to support and strengthen medical radiation protection across Europe.

In 2012, the Bonn Call for Action identified measures to strengthen radiation protection in medicine worldwide. This Call and the necessity for EU Member States to transpose into their legislation the 2013 European Council Directive, laying down safety standards for the protection against dangers arising from exposure to ionising radiation (2013/59/EURATOM), incited the creation of the initiative in 2014.

EuroSafe Imaging, its multidisciplinarity and working groups, has enabled the ESR to take a step further in radiation protection and is a model for other “safe” campaigns. Over the last 6 years, it has aimed to significantly contribute to achieving the goals set out in the Bonn Call for Action, by adapting it to the European perspective and supporting ESR members with the implementation of the European Council Directive. It has disseminated and developed measures for radiation protection such as guidelines, the development of clinical diagnostic reference levels (DRLs), policies and performance indicators. Furthermore, EuroSafe Imaging has worked towards the best possible patient care through the implementation of a clinical audit tool, training and e-learning for patients and professionals. It seeks improved communication with patients about radiological procedures, benefits and risks. The initiative promotes research but also has a network of imaging facilities worldwide that embody best practice. EuroSafe Imaging collaborates with stakeholders, regulatory authorities for a safety culture in medical imaging, as well as industry to ensure up-to-date equipment.

In the future, the EuroSafe Imaging Initiative will continue to raise awareness through its participation in meetings and conferences around the world, social media communication and increased collaborations with major stakeholders.

## Main messages

- The EuroSafe Imaging Call for Action 2018 continues to guide the agenda of radiation protection.
- Over the last six years, EuroSafe Imaging has established a global reach in its efforts to protect patients and imaging stakeholders.
- EuroSafe Imaging has participated in over 140 meetings and conferences worldwide and its EuroSafe Imaging Stars network consists of 128 imaging centres.

## Introduction

The use of ionising radiation is an essential and well-established practice in medicine, both in diagnosis and treatment. However, while the medical benefits are unquestionable, there is also the potential harm of ionising radiation. In addition, there is increasing evidence of unnecessary and occasionally unintentional exposure to radiation. This has given rise to the need for more efforts to better radiation protection in medicine.

In recent years, over 67 million CT scans have been performed per annum in Europe [1]. The increasing use of new imaging techniques, both for diagnosis and treatment like interventional procedures and image guided therapies, has raised a number of issues in the radiological protection of patients and medical staff.

### The background to the ESR EuroSafe Imaging initiative

The European Council Directive 2013/59/Euratom (BSSD) [2] repealing five older European Directives had to be transposed into the national legislation of all EU Member States by 6th of February 2018. The Directive emphasises the need for justification of medical exposures (including those of asymptomatic individuals). Furthermore, it introduces requirements concerning patient information and strengthens those for recording and reporting doses from radiological procedures. It also reinforces the importance of the implementation of diagnostic reference levels, the availability of dose-indicating devices and the improved role and support of medical physics experts in procedures based on ionising radiation, especially some of the imaging procedures with a relevant exposure.

On a global level, the International Atomic Energy Agency (IAEA) and the World Health Organization (WHO) held the *International Conference on Radiation Protection in Medicine: Setting the Scene for the next Decade* in 2012. This led to the formulation of the Bonn Call for Action [3], which identified the main actions essential to strengthening radiation protection in medicine.

The Bonn Call for Action in turn inspired the creation of EuroSafe Imaging in 2014. This led to the first EuroSafe Imaging Call for Action later that year, which adapted the Bonn Call to a European perspective, focusing on developing guidance and tools to support European Society of Radiology (ESR) members with the implementation of the European BSSD (2013/59/Euratom) [4].

The Bonn Call for Action was strengthened at a follow-up conference in 2017 [5], which reviewed the approach to the implementation of the Bonn Call’s actions and looked at how international organisations and other stakeholders could harmonise their actions for better impact. Additionally, it allowed stakeholders to discuss new developments affecting radiation protection in medicine. In response to this, EuroSafe Imaging updated its own Call for Action in 2018. The EuroSafe Imaging campaign’s key role in providing guidance and tools for the implementation of this new legislation has seen its profile rise commensurately.

### Introducing EuroSafe Imaging

**Fig. 1:**
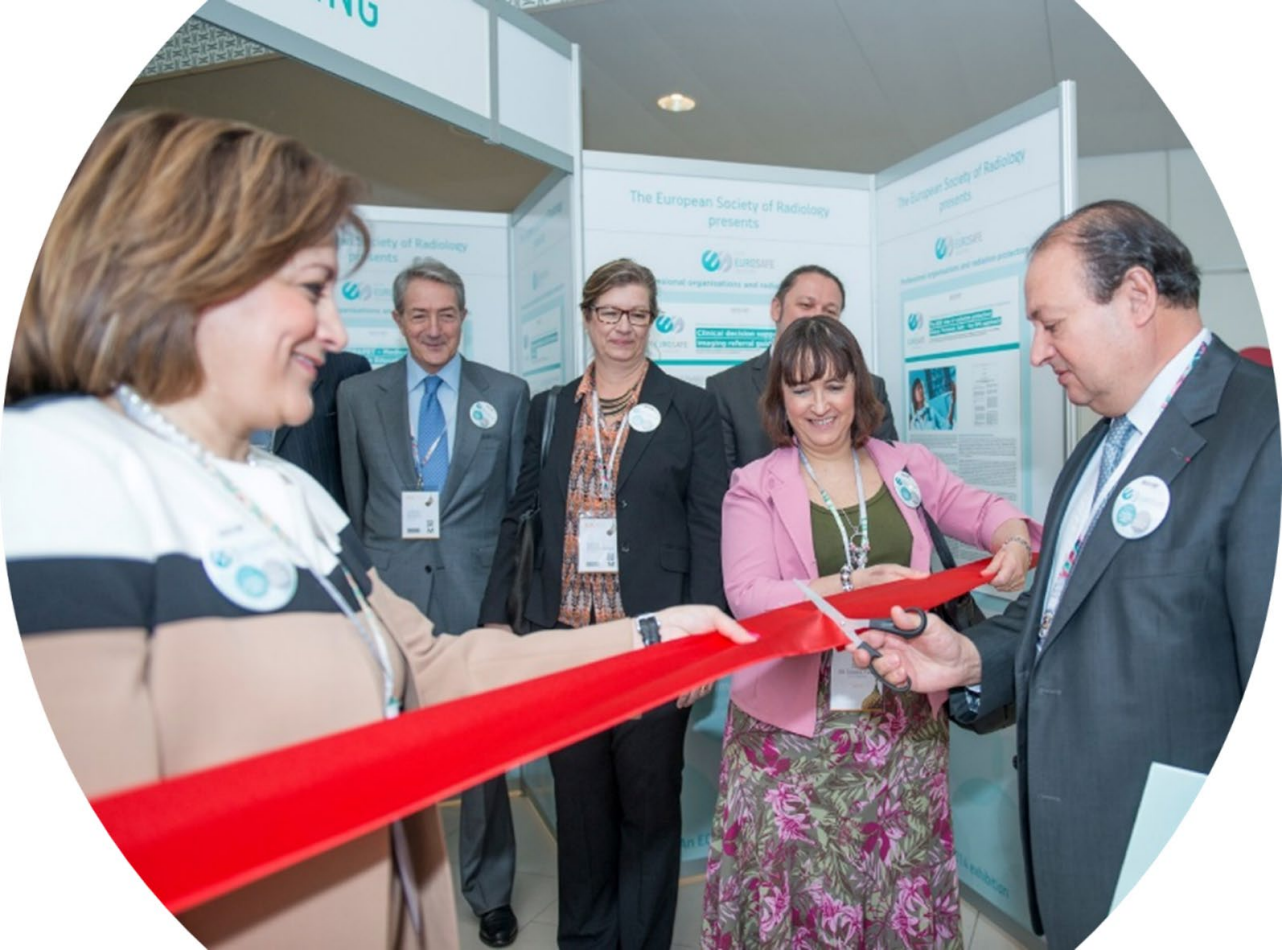
Grand opening of the EuroSafe Imaging area during ECR 2014. From left to right: Nicole Denjoy (COCIR), Lorenzo Bonomo (ESR), Katrine Ricklund (ESR) Georgi Simeonov (European Commission), Maria Perez (WHO), Guy Frija (ESR President)

EuroSafe Imaging’s aim is to promote quality and safety in medical imaging through its mission to support and strengthen medical radiation protection across Europe following a holistic, inclusive approach, collaborating with health professionals and other relevant stakeholders (Fig. 1).

Prior to launching EuroSafe Imaging, the ESR had already led or taken part in several European Commission (EC) tender projects in the area of medical radiation protection. Furthermore, the ESR had worked with major international organisations like the EC, IAEA, WHO, United Nations Scientific Commission on the Effects of Atomic Radiation (UNSCEAR) and the International Commission on Radiological Protection (ICRP). The ESR’s experience of collectively approaching the implementation of radiation protection is a key component in helping the EuroSafe Imaging initiative achieve its vision of safe imaging for patients.

EuroSafe Imaging’s Steering Committee is charged with planning, coordinating, and overseeing the implementation of EuroSafe Imaging’s activities. The Steering Committee is composed of delegates from the ESR, subspecialty societies, related medical professions (medical physicists and radiographers), patient groups and industry, along with observers from the EC, IAEA, WHO and the ICRP Committee 3. This ensures a multi-disciplinary and holistic approach to radiation protection.

The work of EuroSafe Imaging is carried out in working groups: Strategies for Image Quality, Artificial Intelligence (AI) and Research; Dosimetry for Imaging in Clinical Practice; Dose Management; Ask EuroSafe Imaging; Paediatric Imaging; Training Activities; Justification; ESR iGuide Implementation and Promotion; and Clinical DRLs. The EuroSafe Imaging working groups, activities and priorities are presented in detail below as part of the relevant items in the EuroSafe Imaging Call for Action.

EuroSafe Imaging features prominently at every European Congress of Radiology (ECR), showcasing its activities and providing a space for networking in a dedicated lounge. The EuroSafe Imaging poster exhibition has continually grown, with a cumulative total of over 500 posters submitted to date, which are free to view through the EuroSafe Imaging website.

The ECR 2019 marked the fifth anniversary of EuroSafe Imaging [6]. Stakeholders came together to acknowledge five years of hard work to put medical radiation protection on top of the agenda worldwide and celebrate the tangible successes of the initiative. To date, the campaign has attracted the support of more than 68,000 'Friends of EuroSafe Imaging', including over 300 institutional supporters. EuroSafe Imaging has also participated in over 140 international congresses and meetings and has become a role model for the creation of quality and safety campaigns worldwide and as such contributed significantly to the visibility and positive image of the ESR with international organisations and other key stakeholders around the world. Following the inspiration set by EuroSafe Imaging, AFROSAFE and Canada Safe Imaging were founded in 2015. In 2016, they were joined by LATINSAFE in Latin America and the Japan Safe Radiology initiative. In 2017, ArabSafe was launched.

**Fig. 2:**
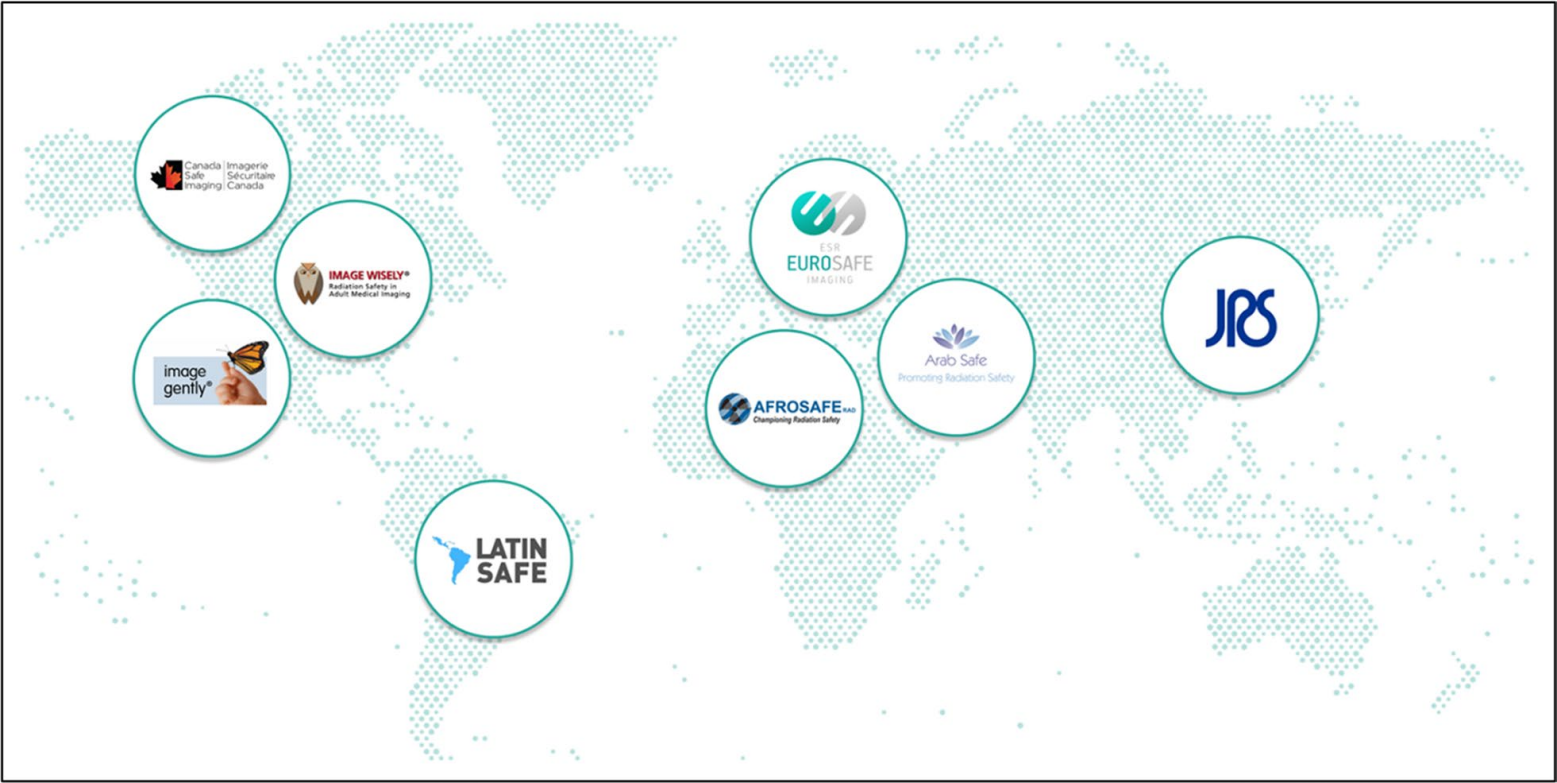
International Society of Radiology Quality and Safety Alliance

All the ‘safe’ campaigns operate under the umbrella of the International Society of Radiology Quality and Safety Alliance (ISRQSA), which consists of the above quality and safety campaigns, plus the US initiatives Image Gently and Image Wisely. The ISRQSA is co-chaired by the EuroSafe Imaging Steering Committee Chair and by the Chair of Image Gently and works in collaboration with other relevant organisations in the domain of safety and quality, including the IAEA, WHO and ICRP. The overarching objective for the ISRQSA in the area of radiation safety is to establish a strategic plan for global efforts related to quality and safety (Fig. 2).

### EuroSafe Imaging and its renewed Call for Action in 2018

**Fig. 3:**
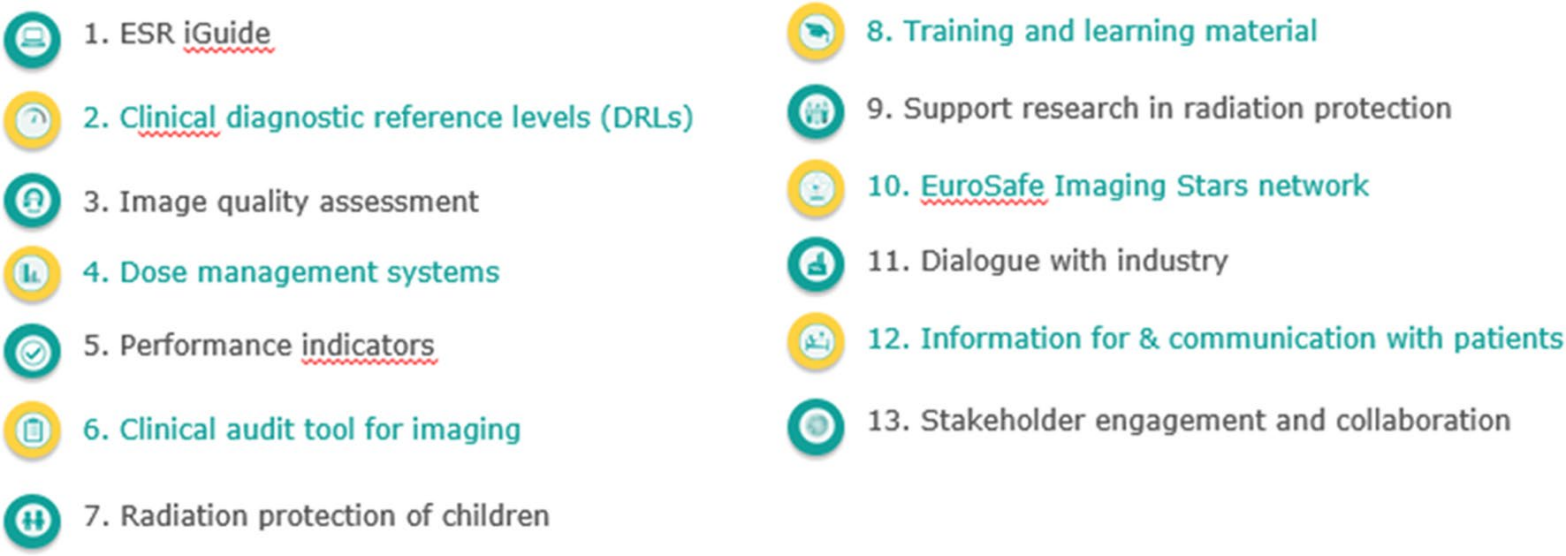
EuroSafe Imaging Call for Action 2018

The EuroSafe Imaging Call for Action 2018 drives the agenda and initiatives of EuroSafe Imaging. By implementing the following 13 actions, EuroSafe Imaging aims to significantly contribute to achieving the goals of the IAEA/WHO’s ten priority areas set out in the Bonn Call for Action (Fig. 3).

### Action 1: Disseminate guidelines and develop implementation policies for a clinical decision support system (ESR iGuide) in Europe

It has long been clear that diagnostic imaging is over-utilised and many imaging referrals are inappropriate, unclear or inconsistent and guidelines are often not used in practice even when they are available to the physicians. One reason for over-utilisation of imaging is described with the term "defensive medicine". This means that imaging procedures are requested increasingly not only for the benefit of the patient, but for the protection of the physician. Evidence-based referral guidelines in clinical practice embedded through decision support systems (such as the ESR iGuide) are intended to improve the use of guidelines by physicians. Activities of the ESR iGuide Implementation and Promotion Working Group,^1^ launched at the ECR 2018, include: developing scenarios for implementation and promoting the use of the ESR iGuide; involving European national radiological societies in the implementation process; and contributing to the dissemination of and supporting the use of the ESR iGuide in low- and middle-income countries.

### Action 2: Develop clinical Diagnostic Reference Levels (DRLs) for adults and children

The ESR was awarded the EC funded EUCLID project (European Study on Clinical DRLs for X-ray Medical Imaging) that started in August 2017 and was completed in June 2020. The main goals were: (i) to collect data needed to establish DRLs for the most important x-ray imaging tasks in Europe with respects to radiation protection; (ii) to stipulate up-to-date DRLs for these clinical tasks; and, to convene a workshop to discuss the project’s results, differing perspectives on DRLs, and how DRLs will affect clinical practice. Following the successful completion of the EUCLID project, EuroSafe Imaging intends to advocate for a similar project for paediatric patients. A EuroSafe Imaging working group on Clinical DRLs has been established to continue the work of the EUCLID project (Table 1).

**Table 1:** EUCLID project key points

EUCLID project key points:
Project started in 2017 with the goal of providing up-to-date clinical DRLs for the most important, from a radiological perspective, x-ray imaging tasks in Europe
A list of 10 clinical indications for CT and 4 clinical indications for IR was agreed with the EC and a group of 19 hospitals from 14 countries around Europe was established to gather data
Results to be published as part of the EC’s Radiation Protection Series in the near future
Related articles:
- Diagnostic Reference Levels based on clinical indications in computed tomography: a literature review [7]
- Radiation dose and diagnostic reference levels for four interventional radiology procedures: results of the prospective European multicentre survey EUCLID (submitted to European Radiology)

### Action 3: Develop image quality assessment based on clinical indications.

As different radiological imaging tasks require different levels of image quality, image data of the highest quality achievable is not necessarily essential for confident diagnosis. Standardised, indication-specific clinical criteria are necessary to objectively judge required image quality. The EuroSafe Imaging Appropriate Image Quality Working Group has, since its launch in 2016, been active in identifying indication-specific clinical criteria and compiling them into sets suited for the assessment of image quality of radiological examinations. In 2019, it was decided to expand the scope of the working group to include "Artificial Intelligence" (AI), thus renaming the Working Group to “Strategies for Image Quality of the Future”. Promotion of Research in radiation protection was also included in this working group.

Similarly to action 2, it was possible to secure research funding from the EC as part of the so called MEDIRAD project. This project involves a clinical based study to determine subjective and objective image quality criteria of chest CT imaging. This study will be based on a new definition of clinical image quality criteria for chest CT that has already been developed.

A paper about the different ways to determine image quality in clinical configurations is under preparation by the above-mentioned working group. It will summarise Fourier- based approaches on imaged phantoms, in clinical images, different approaches for subjective image quality assessment, as well as task-based measures and approaches using model observers.

### Action 4: Promote dose management systems to establish local, national, and European DRLs

The EuroSafe Imaging Working Group on Dose Management, established in 2019, aims to promote dose management systems to establish local, national and European DRLs, as well as requirements for and implementation of dose management software. Dose management plays a more and more important role in quality assurance, optimisation and detection of unintended exposures or overexposures.

A recently published EuroSafe Imaging paper on dose management [8] outlines the following clinical radiation protection tasks supported by DMS:

- Collecting dosimetric data to establish local or national DRLs
- Checking compliance with DRLs
- Prevention, detection and helping in reporting of unintended exposures
- Optimisation of patient exposure, especially in the field of computed tomography (CT) and interventional radiology (IR)
- Structured consolidation of dose documentation, reporting and tracking
- Notification if local or national alert levels are exceeded
- Local, regional or national benchmarking of patient exposure for modalities and procedures

The Working Group on Dosimetry for Imaging in Clinical Practice was established at ECR 2019 with the mission to obtain a European consensus on the concept of dosimetry, including patient and (when appropriate) occupational dosimetry. The group produced the paper ‘Harmonisation of imaging dosimetry in clinical practice: practical approaches and guidance from the ESR EuroSafe Imaging initiative’ [9] published in March, 2020 (Table 2).

**Table 2:** Highlighting the initial topics selected by the Dosimetry for Imaging in Clinical Practice Working Group and the agreed priorities for these topics as outlined in the paper ‘Harmonisation of imaging dosimetry in clinical practice: practical approaches and guidance from the ESR EuroSafe Imaging initiative’

Topic	Agreed priority/response
Information on patient exposure for patients	The preferred option for the majority of experts was to inform on “the dose values and units, reported by the X-ray system”
Individual optimisation	The majority of experts highlighted the following aspect “Consider patient and staff doses for interventional procedures”
Accidental and unintended exposures	All the experts agreed with the following priority “If suspected an accidental or unintended exposure, record and analyse the dose parameters (based on physical quantities) and produce a report for the quality assurance committee”
Dosimetric trigger levels (for individual procedures)	All the experts agreed with the following priority “Trigger levels should be established for interventional procedures to alert on the risk of potential skin injuries”
Comparison with Diagnostic Reference Levels (DRLs)	All the experts agreed with the following priority “The comparison with DRLs should be made at least, once per year and after changes in the X-ray unit or in the imaging protocols”
Role of the dose registry and management systems	All the experts agreed with the following priority “These systems should allow fulfilling the regulatory requirements (directive 2013/59/EURATOM) on patient dose registration”
Dosimetric information for the practitioner	All the experts agreed that the practitioner should have information on the physical quantities offered by the X-ray system for the different imaging modalities
Dosimetric information for the referrer	For this question, there was no agreement between the experts for the options offered (physical dosimetric quantities, effective doses, or diagnostic reference levels). This aspect would need further discussion

### Action 5: Develop performance indicators for radiation protection management

To support the prevention of medical radiation incidents and accidents, and to strengthen radiation safety culture, improvements in quality management tools are required. The EuroSafe Imaging Steering Committee and ESR Subcommittee on Professional Issues and Economics in Radiology (PIER) have analysed existing quality management tools in radiation protection, developed a catalogue of key performance indicators and prepared and presented material to promote such indicators. Continuous recording of such indicators can be used as a basis for a dashboard with values, or to act as a warning system. A EuroSafe Imaging paper on this topic was published [10].

### Action 6: Implement a clinical audit tool for imaging to improve the quality of patient care

**Fig. 4:**
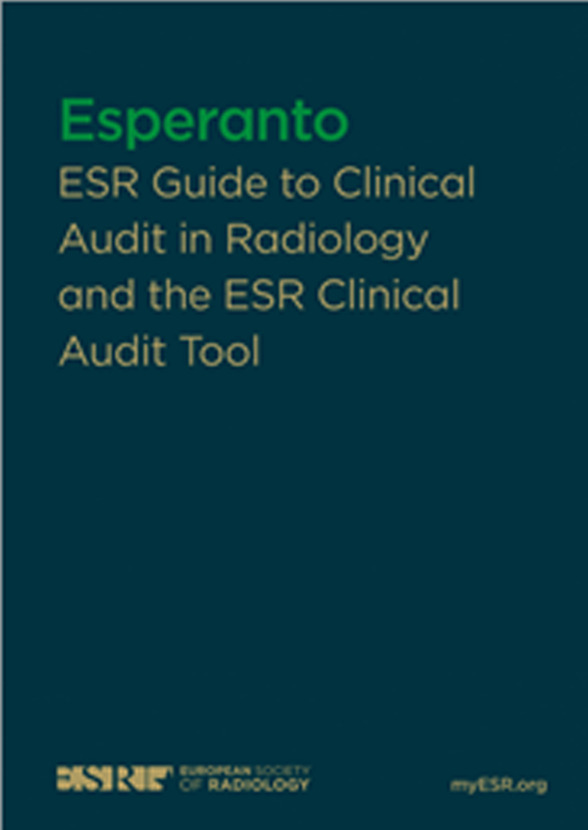
Esperanto booklet

The EU BSS Directive [2] requires clinical audits to be carried out “in accordance with national procedures.” In this context, the ESR Audit and Standards Subcommittee has developed the Esperanto Booklet - a guide to clinical audit in radiology and the ESR clinical audit tool - to support imaging departments in developing a clinical audit programme. Internal clinical audit helps departments to comply with legislation, monitor their own practice and to be well prepared for any external audit. This booklet provides an outline of the principles of clinical audit and a library of templates for audit in various situations. Surveys among EuroSafe Imaging Star Departments and ESR National Member Societies have been carried out to evaluate and monitor the status of clinical audit practice across Europe and measure awareness of the ESR Esperanto Booklet [11, 12] (Fig. 4; Table 3).

**Table 3:** Esperanto booklet audit topics

Regulatory audit topics (relating to regulation of medical exposures using ionising radiation)
(1) Is there a departmental mechanism for providing patients (or their representative) with information relating to the risks/benefits associated with radiation dose from the medical exposure?
(2) Is there an established mechanism within the department to register and analyse accidental /unintended exposures?
(3) Is there a departmental policy for informing patients, or their representative, that they have undergone an accidental exposure?
(4) Is there a mechanism for record keeping and retrospective analysis of accidental or unintended medical exposures?
(5) Is there a mechanism for referring accidental exposure events to the medical physics expert (MPE) and informing the competent authority of significant events?
(6) Does the department utilise criteria, provided by the relevant radiation protection competent authority, for what constitutes an accidental or unintended significant exposure?
(7) Is there evidence for appropriate training for individuals with delegated responsibility (in the case of nonradiologists) for the justification process?
(8) Is there a departmental mechanism to confirm and document the non-pregnancy status of individuals undergoing medical exposures?
(9) Is there a written protocol for the identification of who is responsible for the justification process?
(10) For radiation exposure related to health screening by invitation on asymptomatic individuals, is there a local policy affirming justification by a competent authority?
(11) What percentage of examinations involving ionising radiation are justified in advance of being performed?
(12) What mechanism exists on the request form for contacting referrers to permit pre-exposure justification discussions to occur if necessary?
(13) Is there a written protocol for who may be responsible for justification of X-ray/fluoroscopic/ interventional ionising radiological procedures?
(14) Is there a written protocol for who may be responsible for justification of CT examinations?
(15) What mechanism is used to evaluate patient dose in high-dose procedures?
(16) What percentage of radiodiagnostic procedures have established diagnostic reference levels (DRL)?
(17) Specific technical requirements for equipment in use for medical exposures
(18) Eye lens dose limits for occupational exposure
(19) Initial education and training in radiation protection
(20) Audit of education plus training in radiation protection, doses and side effects
(21) Provision of clinical information to support justification
(22) Staff dosimetry audit – this includes a draft adapted questionnaire
(23) Evaluation of the role and responsibilities of the medical physics expert

Clinical audit topics (relating to service provision and clinical practice)
(1) Does the radiology department record statistics about patient satisfaction?
(2) Waiting time for outpatient ultrasound appointments
(3) Protocols around radiological procedures, information in reports
(4) The practice of “routine” preoperative chest x-ray
(5) Audit of inpatient chest x-rays or abdominal x-rays
(6) What percentage of non-Ionising imaging studies (MR/Ultrasound) are consistent with the referral guidelines?
(7) Pain sensation during image-guided interventions

An ESR-led consortium, with EANM and ESTRO, was awarded the EC tender QuADRANT. This 30-month project started in January 2020 and aims to promote constant improvement in quality and safety of radiology, radiotherapy and nuclear medicine, through the implementation of clinical audit.

### Action 7: Radiation protection of children: develop guidance for good and safe use of imaging, and for effective communication

Children are particularly at risk of harm from non-justified studies or studies with non-optimised protocols due to their increased sensitivity to ionising radiation exposure and longer life expectancy. Children were the focus of EuroSafe Imaging during ECR 2020. Paediatric Imaging was a focus of the EuroSafe Imaging Poster Exhibition and a flyer on Medical Radiation Protection of Children was produced.

The European Commission issued Radiation Protection 109 (RP 109), ‘Guidance on diagnostic reference levels (DRLs) for medical exposure’ in 1999, highlighting the importance of DRLs for high-dose medical examinations of patients sensitive to radiation, especially children. The EC recognised the need to consolidate existing DRLs and to provide guidance for using DRLs to further enhance the radiation protection of children by approving the 27-month European DRLs for Paediatric Imaging (PiDRL) tender project in December 2013. The specific objectives were to: agree on a methodology for establishing and using DRLs for paediatric imaging; and, update and extend the European DRLs to cover more procedures and a wider patient age/weight-range based on current knowledge [13].

The EuroSafe Imaging Paediatric Imaging Working Group has submitted a paper based on the results of a survey to assess the awareness, availability and use of referral guidelines for medical imaging in children among European radiologists.

### Action 8: Organise radiation protection training courses and develop e-learning material to promote a safety culture and raise awareness on radiation protection

To improve information for professionals and patients, the ‘Ask EuroSafe Imaging’ initiative comprised of dedicated working groups; these working groups comprise of one radiologist, one medical physicist and one radiographer in order to ensure comprehensive and well-balanced content. The initiative has produced ‘Tips & Tricks’ in CT, interventional radiology and paediatric imaging [14], all of which are freely accessible on the EuroSafe Imaging website (Table 4). Other radiation protection educational opportunities include: EuroSafe Imaging webinars^2^; the ESR’s Education on Demand platform that offers e-learning modules; and the EuroSafe Imaging checklist ‘Managing a Safe CT service’, available in English, Portuguese and Spanish. Finally, the ESR eGuide, a new e-learning project utilising the ESR’s iGuide decision support portal, has been launched, tying in actions 1 and 6.

**Table 4:** Tips & Tricks editions published in 2019-2020

CT Tips & Tricks	IR Tips & Tricks	Paediatric Tips & Tricks
Iterative reconstruction algorithms	Checklist modification	Safety and dose aspects of prenatal medical irradiation
Automated tube voltage	Medical Simulators for Training in Dose Management and Radiation Protection	Special aspects when using dose quantities in paediatric radiology
To shield or not to shield?	Hand protection for interventionalists	Interacting with children during radiographic procedures
Ultra-low dose emergency chest CT	Eye protection for interventionalists	Referral guidelines for diagnostic imaging in children: ESR iGuide
Dose saving options in maxillofacial trauma	Eye Dose Limits Achieving Regulatory Compliance in Interventional Radiology	Dose-saving technologies in paediatric radiology
First aid kit for complying with the radiation protection requirements in a CT ward	How to reduce the dose in Paediatric Interventional Radiology	Producing information on diagnostic imaging examinations for children, young people, and families
Breast Bismuth Shields: Should be used?	Pregnant workers in interventional radiology	Adaptation of protocol parameters to paediatric patients

### Action 9: Support research in advanced topics of radiation protection, e.g. artificial intelligence, and facilitate the dissemination and translation into clinical practice of this research

**Fig. 5:**
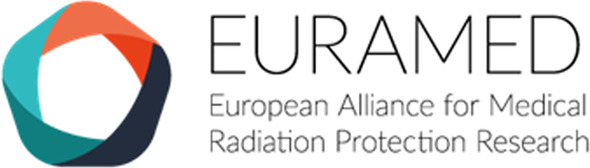
Euramed logo

The high-quality radiation protection we enjoy in clinical practice today is built upon many years of research by healthcare professionals. The European Alliance for Medical Radiation Protection Research (EURAMED) has defined the research topics considered most necessary for effective medical care and most efficient in terms of radiation protection. EuroSafe Imaging is committed to promoting EURAMED’s strategic research agenda and helping identify research priorities (Fig. 5).

It is envisaged that for 2020 the Image Quality Working Group will feature a dedicated subset on radiation protection research to provide coordination for the ESR contribution to EURAMED’s activities. The EURAMED rocc-n-roll EU tender project, led by EIBIR, starting in 2020 is aimed at providing an updated and comprehensive strategic research agenda and an active EuroSafe Imaging role is planned in order to adapt it to Radiology.

### Action 10: Strengthen the EuroSafe Imaging Stars network of imaging centres that embody best practice in radiation protection

EuroSafe Imaging Stars, launched in 2016, is a scheme designed to identify and recognise imaging facilities worldwide that embody best practice in radiation protection. By partnering with institutions that are committed to putting the principles advocated and concepts developed by the European Society of Radiology into practice, EuroSafe Imaging aims to bridge the gap from raising awareness and advocacy to impacting the reality of clinical practice.

**Fig. 6:**
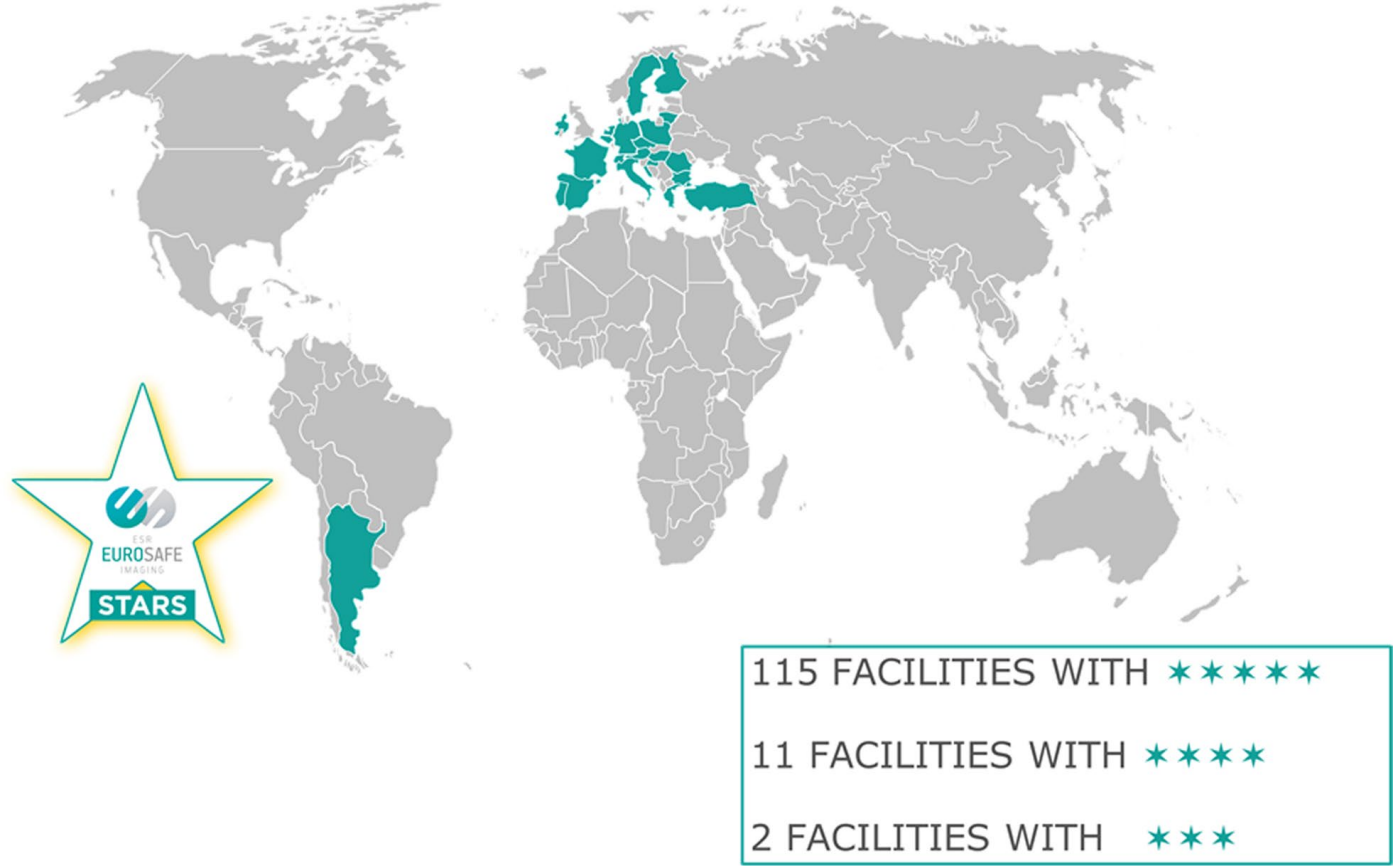
Map of the EuroSafe Imaging Stars network

As of April 2020, 128 EuroSafe Imaging Stars have been awarded across the world. A new application form is being prepared in order to better consider the BSSD requirements in the application process (Fig. 6).

### Action 11: Establish a dialogue with industry regarding improvement of radiological equipment, the use of up-to-date equipment (e.g. dose management systems), and the harmonisation of exposure indicators

Manufacturers play a key role in medical radiation protection as they are responsible for developing the equipment used in imaging departments. EuroSafe Imaging is lobbying for the improvement and use of up-to-date equipment, and will campaign for the establishment of a European plan supported by the EC. The EuroSafe Imaging Steering Committee includes a representative of COCIR, the European Coordination Committee of the Radiological, Electromedical and Healthcare IT Industry. In addition, an informal dialogue group between EuroSafe Imaging and COCIR was established with the aim of discussing concerns and issues relating to the implementation of the BSS Directive from an industry perspective.

### Action 12: Improve information for and communication with patients about radiological procedures, related benefits and possible risks

Adequate patient information and effective risk-benefit communication are still frequently lacking in imaging departments. In a pioneering role, the ESR’s Patient Advisory Group is represented on the EuroSafe Imaging Steering Committee in order to give patients a voice. EuroSafe Imaging advocates for the improvement of existing, and development of new, patient information such as the ‘What Patients Should Know’ publications [15], developed by the Ask Eurosafe Imaging groups.

### Action 13: Engage with stakeholders and collaborate with related initiatives and regulatory authorities in Europe and beyond to contribute to a global safety culture in medical imaging

EuroSafe Imaging is the ESR’s platform to engage with decision makers, including the IAEA, WHO and Heads of the European Radiological protection Competent Authorities (HERCA). In February 2017, the ESR and the IAEA concluded a practical arrangement on capacity building, education and training in the area of diagnostic imaging including hybrid imaging, building on the longstanding cooperation between the ESR and the IAEA, particularly its Nuclear Medicine and Diagnostic Imaging Section. Concrete topics of cooperation include the ESR iGuide and the EuroSafe Imaging Stars concept and the establishment of centres of reference.

ESR supported HERCA in the preparation of a 2019 campaign to raise awareness of the implementation of the justification principle in diagnostic radiology among referrers and regularly invites speakers to EuroSafe Imaging sessions at ECR.

**Fig. 7:**
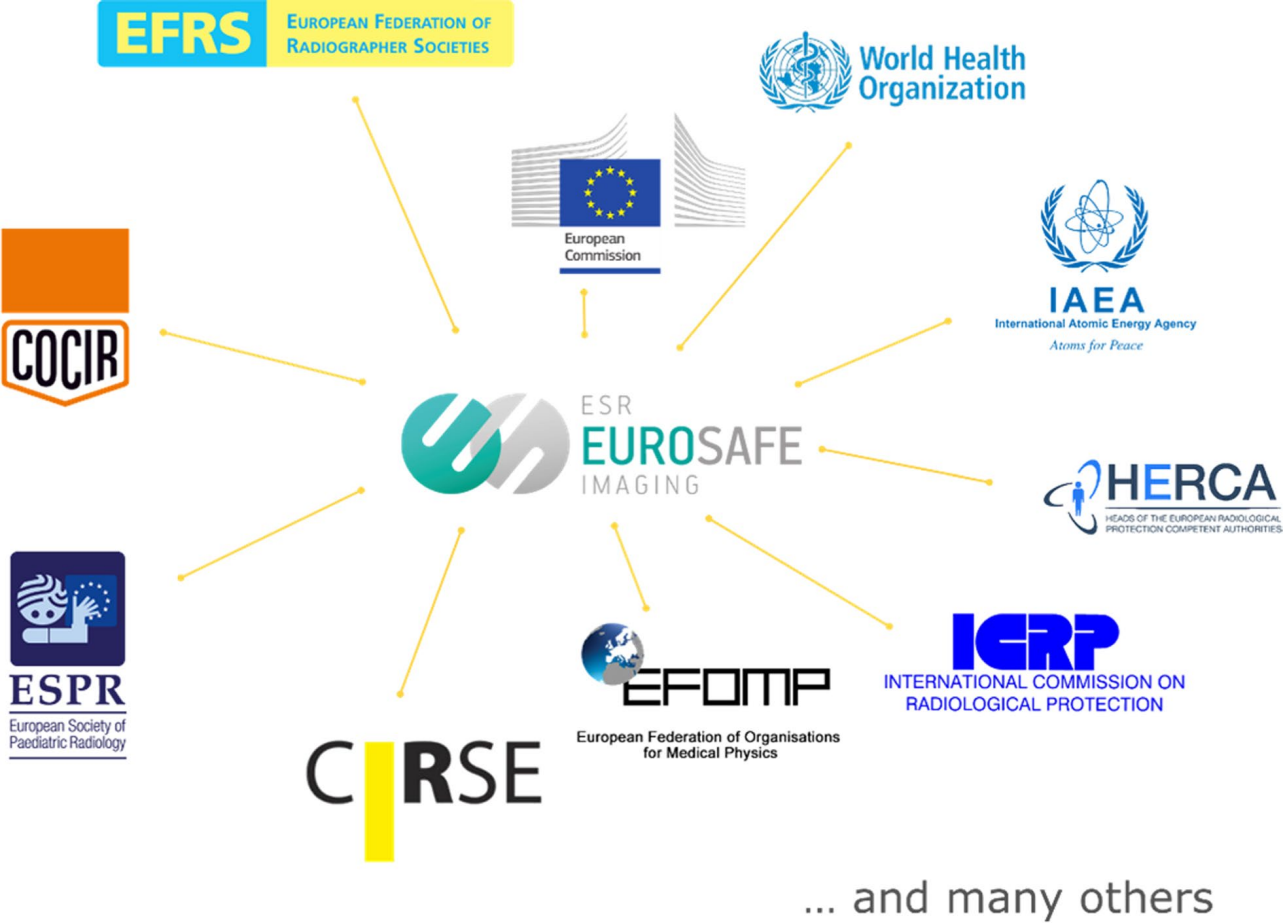
EuroSafe Imaging collaborators and stakeholders

Throughout all of the above-mentioned projects, there are interlinks with stakeholders, patients and regulatory authorities in Europe as well (Fig. 7).

## EuroSafe Imaging looking forward

Communication and outreach play a pivotal role in EuroSafe Imaging’s mission to strengthen and support quality and safety in medical imaging. EuroSafe Imaging seeks to raise awareness through its participation in meetings and conferences around the world. The campaign also recently extended its communication channels by establishing dedicated Twitter and Facebook accounts, providing regular updates on the campaign’s activities.

EuroSafe Imaging is developing a radiation protection webinar programme, helping to ensure a high standard of knowledge in the field. Increasing collaboration with IAEA will play a major role, in particular regarding efforts to expand the EuroSafe Imaging Stars and to make ESR iGuide available to low- and middle-income countries.

Finally, to support implementation of the justification requirements in the BSSD, a Justification Working Group has been established with a paper planned for publication in 2020.

## Summary

Over the last six years, EuroSafe Imaging has achieved significant success in radiation protection and has established a global reach in its efforts to protect patients and imaging stakeholders. Its collaborations with international organisations give it a powerful advocacy role. The EuroSafe Imaging Call for Action continues to guide the agenda of radiation protection. It can only be hoped that the next years continue to bring success on such a broad scale and to achieve this, the contribution of many more, especially young professionals, in the field of radiation protection is of upmost importance.

## Data Availability

All data and material are included in this article.

## Footnotes

1 A full list of EuroSafe Imaging’s Steering Committee and all its current working groups can be found here: http://www.eurosafeimaging.org/about/steering-committee/eurosafe-imaging-working-groups.

2 The ESR has an ongoing collaboration on issues of common interest with IAEA e.g. the successful collaboration with the IAEA’s Radiation Protection of Patients Unit for the webinar ‘Radiation protection in interventional radiology: practical hints and tricks’, which took place on 6th Sept 2018.
